# Accuracy, feasibility and predictive ability of different frailty instruments in an acute geriatric setting

**DOI:** 10.1007/s41999-022-00645-1

**Published:** 2022-04-23

**Authors:** Rafael Bielza, Cristina Balaguer, Francisco Zambrana, Estefanía Arias, Israel J. Thuissard, Ana Lung, Carlos Oñoro, Patricia Pérez, Cristina Andreu-Vázquez, Marta Neira, Noemi Anguita, Carmen Sáez, Eva María Fernández de la Puente

**Affiliations:** 1 Department of Geriatric Medicine, Hospital Universitario Infanta Sofía, Paseo de Europa 37, 28007 San Sebastián de los Reyes, Spain; 2 Department of Medicine, Faculty of Biomedical and Health Sciences, Universidad Europea de Madrid, Madrid, Spain; 3 Department of Geriatric Medicine, Hospital Central de la Cruz Roja San José y Santa Adela, Madrid, Spain; 4 Department of Oncology, Hospital Universitario Infanta Sofía, San Sebastián de los Reyes, Madrid Spain

**Keywords:** Frailty, Accuracy, Feasibility, Acute care setting

## Abstract

**Purpose:**

To analyze the feasibility, accuracy and the ability of different frailty instruments to predict adverse outcomes.

**Methods:**

A prospective cohort study was conducted in patients ≥ 70 years admitted to the acute care setting (ACS). Feasibility and prevalence of frailty were assessed by FRAIL, Clinical Frailty Scale (CFS), hand grip strength (HGS) and the Spanish Frailty-VIG. Receiver operator characteristic (ROC) curves and area under the curve (AUC) were performed to identify frailty according to each instrument, setting VIG as the reference. For each instrument, multiple logistic regressions were used to examine the effect of frailty on primary outcome (i.e., three-month mortality) and secondary outcomes (i.e., in-hospital mortality, length of stay, institutionalization, functional decline and 30-day readmission).

**Results:**

A total of 185 patients were included, with a median age of 89 years. The feasibility of the instruments was 100%, except for HGS (67%). The prevalence of frailty varied from 65.2% (FRAIL) to 86.7% (VIG). AUCs against VIG ranged from 0.69 (95% confidence interval [CI] 0.57–0.81: FRAIL) to 0.77 (95% CI 63.5–90.2: CFS). Frail patients defined by FRAIL were 2.7times more likely to have a prolonged length of stay than non-frail patients (95% CI 1.385–5.416). Three-month mortality occurred more among frail patients, either defined by FRAIL (OR 2.5; 95% CI 1.072–5.881) or CFS (OR 3.7; 95% CI 1.255–10.812), than in non-frail patients.

**Conclusion:**

The four instruments had high feasibility providing variable prevalence of frailty. FRAIL and CFS predicted well for three-month mortality, and FRAIL also for length of stay. However, none of the instruments predicted for the other secondary outcomes of the study.

**Supplementary Information:**

The online version contains supplementary material available at 10.1007/s41999-022-00645-1.

## Key Summary Points

**Aim:**

To investigate the feasibility and accuracy of four frailty instruments: FRAIL, Clinical Frailty Scale (CFS), hand grip strength (HGS) and the Spanish Frailty-VIG; and to evaluate their ability to predict adverse outcomes in an acute care setting (ACS).

**Findings:**

The four instruments had high feasibility but provided variable prevalence of frailty. FRAIL and CFS predicted well for three-month mortality, and FRAIL also for length of stay. However, none of the instruments predicted for the other outcomes.

**Message:**

The FRAIL and CFS may be of value in diagnostic and therapeutic decision-making in an acute geriatric setting, given their prognostic ability and feasibility. Further studies are needed to identify the best frailty instrument in an ACS.

**Supplementary Information:**

The online version contains supplementary material available at 10.1007/s41999-022-00645-1.

## Introduction

Frailty is a biological syndrome consisting of a decreased functional reserve caused by the decline of multiple physiological systems, leading to a loss of homeostatic capacity and making individuals more vulnerable to adverse events [1]. The frail older people living in the community have a higher risk of hospitalization, mortality, dependence, falls or institutionalization [2], whereas this condition leads the hospitalized older patients to worse outcomes in terms of in-hospital and long-term mortality, functional decline or institutionalization [3, 4]. There are two complementary views of this syndrome: (1) the phenotypic model of frailty, which is particularly useful for population screening to identify pre-disability states, and, (2) the deficit accumulation approach, that considers frailty as a quantifiable continuum of age-related health deficits [2]. In this regard, the Linda Fried Frailty Phenotype instrument is highly feasible to measure in nursing homes, primary care and outpatient settings frailty according to the phenotypic model, but its feasibility is significantly lower in those admitted to an AGS [5, 6]. The hand grip strength (HGS) as a measurement of muscle strength is a dimension of the Linda Fried Frailty Phenotype along with unintentional weight loss, self-reported exhaustion, sedentary behavior and slow gait speed, and HGS has shown predictive validity for decline in cognition, mobility, functional status and mortality in older people living in the community [7]. On the other hand, the Frail-VIG Index (VIG) is based on the Comprehensive Geriatric Assessment (CGA), measures 22 dichotomic variables to asses deficit accumulation in several domains, and has been recently validated in an AGS in Spain [8]. This and other instruments within deficit accumulation model [9, 10] are long, time-consuming and complex to apply, and therefore simpler and shorter screening instruments such as the Clinical Frailty Scale (CFS) [11] and the FRAIL questionnaire have been developed and are being increasingly used in AGS [12]. The CFS has strong correlation with the VIG and other instruments within the deficit accumulation model, and the FRAIL questionnaire is considered a mixed test as it is composed of four items of physical frailty and one of comorbidity [12, 13].

Frailty instruments are of outmost value in acute geriatric units, as they not only allow the clinician to establish a prognosis, but also to personalize the goals of care and tailor the diagnostic and therapeutic interventions [4]. However, there is no consensus on which frailty instrument is better to be applied at AGS [14]. Consequently, we sought to evaluate four existing instruments with different frailty approaches for the detection of this syndrome in AGS. This study aims to (1) identify the feasibility of these different frailty instruments (HGS, VIG, CFS, FRAIL); (2) compare their accuracy to identify frailty using the VIG as the reference and, (3) evaluate their ability to predict adverse outcomes among hospitalized older adults admitted to an AGS.

## Methods

### Population and study design

The study population consisted of older patients who were consecutively admitted to the Department of Geriatric Medicine at an academic tertiary care hospital, from 1 June 2019 to 31 December 2020 with a follow-up period of three months. This hospital covers a population of 312,000 inhabitants in the north of Madrid where around 500 older patients are attended per year in the acute geriatric wards.

Patients aged 70 and older were eligible for enrollment in the prospective cohort study if they were admitted to the Geriatric acute care wards and provided (themselves or a legal representative) a signed written informed consent document within 24 h of admission. Patients who suffered from COVID-19 within three months of discharge were excluded from the analysis of mortality and readmission rates.

Patients were assisted according to the principles of acute geriatric units, i.e. comprehensive geriatric assessment and care focused on the needs of the patients, interdisciplinary work carried out by a core team of professionals (geriatrician, nursing staff trained in geriatrics and social worker), and early discharge planning [15, 16]. In addition, a geriatrician, a specialist geriatric nurse and a geriatric student were responsible for the administration of the written informed consent document to participants. Data collection and assessment of frailty and comprehensive geriatric assessment were obtained within 24 h of admission. Our research interviewers contacted participants (or their legally acceptable representative) at three months after enrollment to determine their mortality and 30-day readmission.

### Frailty assessment

In supplementary Fig. 1 and supplementary tables 1 and 2, we summarize the main features of the different instruments used to assess frailty in this study. The Rockwood Clinical Frailty Scale (CFS) considers the pre-existing level of function and mobility and classifies patients from (1) very fit to (9) terminally ill based on easy-to-understand pictograms and descriptors. When the score is ≥ 4, the patient is considered frail [17].

The HGS in kilograms (kg) was measured in the self-reported stronger arm using a Jamar Hand Dynamometer, with participants seated in a chair and the higher value of two trials was used for data analysis [7]. We considered as a cut-off point for frailty in men a HGS < 23 kg and < 12 kg in women, according to the normal values from the Frailty and Dependency Study Cohort (FRADEA) conducted in our country [18].

The FRAIL is a short interview-based tool (1–3 min) designed to assess fatigue, endurance, ambulation, weight loss and illness, with score range from 0 to 5. Values ≥ 3 identify the individual as being frail [13].

The Frail-VIG is a multidimensional index based on the accumulation of deficits extracted from the CGA, measuring 22 variables grouped in 8 domains: functional, nutritional, cognitive, emotional, social, geriatric syndromes prior to admission, symptoms with criteria of severity and the presence of chronic diseases. The Index range is from 0 to 1 point, coming from dividing the total sum of the points of the variables into 25, considering individuals with VIG scores ≥ 0.2 as being frail [19]. This instrument has been validated in the Spanish population in patients admitted to acute geriatric wards, contrary to other instruments that in addition are mostly applied in other settings. Therefore, VIG instrument was considered as the reference in this study [8, 19].

Feasibility was assessed on the percentage of patients with all composites of the VIG, CFS, and FRAIL scales completed; if any scale item was missing, it was considered incomplete. In the case of the HGS, feasibility was evaluated on the percentage of patients able to understand and coordinate the action of pressing the dynamometer.

### Other variables of the study

In addition to the aforementioned variables, we also recorded age (years), sex (male or female), ability to perform activities of daily living before admission and at discharge according to the usual cut-off points of the Barthel Index [20], percentage of patients with dementia (considered when the diagnosis of dementia was previously made in an outpatient clinic) [21] and their stages according to the Global Deterioration Scale (i.e., mild-moderate cognitive impairment, equivalent to Global Deterioration Scale 4–5 or severe-very severe cognitive impairment, equivalent to Global Deterioration Scale 6–7) [22], place of residence before admission (i.e. nursing home or community-dwelling) and in-hospital diagnostics, grouped as cardiovascular, digestive, respiratory infections, neurological disorders, nephro-urinary diseases and others.

### Adverse outcomes among hospitalized older adults

The primary outcome measure was three-month mortality. Secondary outcome measures included (1) in-hospital mortality, (2) prolonged length of stay (defined as higher than 6 days), (3) new institutionalization at discharge, (4) functional decline at discharge (defined as a worsening of ≥ 5 points in Barthel Index at discharge compared with premorbid) [20, 23], (5) 30-day readmission and, (6) a composite adverse outcome that combines prolonged length of stay or functional decline or new institutionalization.

### Statistical analyses

Baseline characteristics of the sample were presented as mean values ± standard deviation (SD), or median value and interquartile range (IQR) for continuous variables according to parametric test results, and as absolute and relative frequencies for categorical variables.

To assess the accuracy of frailty classification of each instrument, we derived receiver operator characteristic (ROC) curves for HGS, CFS, FRAIL, using VIG as the reference. A patient was considered to be frail when the VIG score was ≥ 0.2. For each instrument, area under the curve (AUC) and its 95% confidence interval was calculated. An instrument was considered to acceptably diagnose frailty (according to VIG classification) when the AUC was greater than 0.7, and considered unacceptable when AUC was below 0.6 [24]. Additionally, ROC contrasts between each of the frailty instruments were performed to determine if there were statistical differences among AUC. In addition, the maximum likelihood cut-off point between sensitivity and specificity was calculated for each frailty instrument. To assess the effect of being frail, according to the usual definition for each instrument (i.e. CFS > 4, VIG ≥ 0.2, FRAIL ≥ 3 and HGS < 23 kg in men or < 12 kg in women) on the risk for each adverse outcome, multivariate logistic regressions where performed and the effect was adjusted for age, sex and principal diagnoses.

Finally, to discriminate the ability of each frailty instrument to predict adverse outcomes, we analyzed the ROC curves, calculating the AUC and its 95% confidence interval (CI). Frailty scales were considered as continuous variables and HGS was distributed by sex. For each ROC, curves a score greater than 0.7 was considered acceptable predictive ability and below 0.6 as unacceptable [24].

The existence of statistical significance was considered when the p value was less than 0.05. The analysis was performed with IBM SPSS 21.0

### Ethical approval

The study complied with good clinical practice standards set forth in the Declaration of Helsinki of 1975 and was approved by the relevant institutional review board: Ethical and Research Committee of the Hospital.

## Results

### Baseline characteristics and feasibility of the scales

**Fig. 1:**
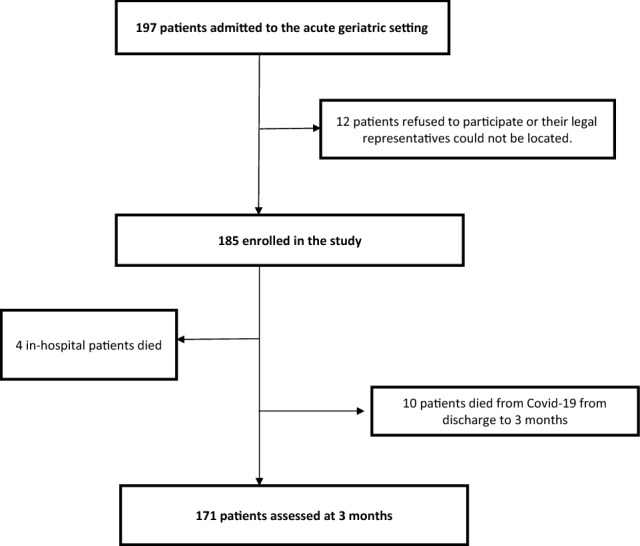
Flow-chart of the study

A total of 185 patients complied with the inclusion criteria of the study, and 171 could be analyzed for three-month mortality (Fig. 1). The median age of participants was 89 [85–93] years, a high percentage of them with dementia (63.8%), mostly in mild stage (43.8%), with a predominance of female participants (60.5%) and the majority was living in the community (60%). The most frequent principal diagnosis was respiratory infections (42.2%), followed by neurological disorders (17.8%) and nephro-urinary diseases (11.4%). The feasibility of the FRAIL, VIG and CFS scales was 100%, whereas in for the HGS it was 67% (Table 1).

**Table 1:** Baseline characteristics of study participants

	*N* = 185
Age, years	89.0 (85.0–93.0)
Sex	
Females	112 (60.5)
Males	73 (39.5)
Prior Barthel index	45.0 (16.3–73.8)
Barthel index at discharge	40.0 (14.4–65.6)
Dementia	118 (73.8)
Place of residence	
Nursing home	74 (40.0)
Community-dwelling	111 (60.0)
Frail VIG index	
Feasibility	185 (100)
Median score	0.4 (0.3–0.5)
< 0.2 (non-frail)	22 (11.9)
≥ 0.2 (frail)	163 (88.1)
Clinical Frailty Scale	
Feasibility	185 (100)
Median score	6.0 (3.0–8.0)
< 4 (non-frail)	47 (25.4)
≥ 4 (frail)	138 (74.6)
FRAIL scale	
Feasibility	185 (100)
Median score	3.0 (2.0–4.0)
< 3 (non-frail)	68 (36.8)
≥ 3 (frail)	117 (63.2)
Hand grip strength	
Feasibility	124 (67.0)
Median score in men	17 (12–22)
Median score in women	10 (6–14)
Frail according to hand grip strength^a^	89 (71.7)
Principal diagnosis	
Cardiovascular	18 (9.7)
Digestive	11 (5.9)
Respiratory infections	78 (42.2)
Nephro-urinary diseases	21 (11.4)
Neurological disorders	33 (17.8)
Others	24 (13.0)
In-hospital mortality	7 (3.8)
Length of stay	6.0 (3.3–8.8)
Functional decline	35 (19.7)
Instituzionalization at discharge	9 (5.1)
Readmitted within 30 days	61 (34.9)
3-Month mortality	47 (25.7)

Results are expressed as *n* (%) or median (Q1–Q3)

^a^Men with a hand grip strength < 23 kg and < 12 kg in women were considered frail

### Accuracy of frailty classification

**Fig. 2:**
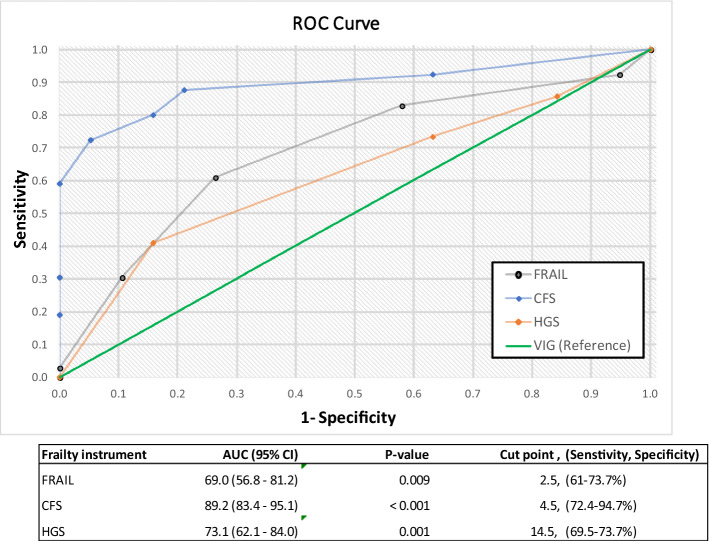
The AUCs for FRAIL, CFS, and HGS against the reference VIG in diagnosis of frailty. *ROC* receiver operator characteristic, *CFS* Clinical Frailty Scale, *HGS* hand grip strength, *AUC* area under the curve

The prevalence of frailty was 88.1%, 74.6%, 63.2% and 71.7%, when assessed using VIG, CFS, FRAIL and HGS, respectively (Table 1). AUCs for FRAIL, CFS and HGS against the reference VIG for diagnosis of frailty were 0.69 (95% confidence interval [CI] 0.57–0.81; *P* = 0.009), 0.89 (95% CI 83.4–95.1; *P* < 0.001) and 0.73 (95% CI (62.1–84); *P* = 0.001), respectively. On ROC contrasts, the AUC was significantly different between CFS vs. FRAIL (*P* = 0.003), whereas we did not find differences between the AUC of FRAIL vs. HGS (*P* = 0.517), and the AUC of CFS vs. HGS (*P* = 0.054) in the detection of frailty, as defined by VIG (Fig. 2). The CFS provided greater sensitivity (72.4%), whereas the CFS provided greater specificity (94.7%) in the diagnosis of frailty.

### Predictive ability of the frailty instruments for adverse outcomes

Regarding negative outcomes, we found an in-hospital mortality of 3.8%, a median length of stay of 6 days (3.3–8.8), 19.7% developed functional decline, 9.1% need institutionalization at discharge, 34.9% were readmitted within 30 days, and 25.7% died within 3 months (Table 1).

Within the multivariate analysis adjusted for sex, age, and principal diagnosis, we found that frail patients (defined by FRAIL) were 2.7 times more likely to have a prolonged length stay than non-frail patients (58.1% vs. 39.7%; 95% CI of the OR: 1.385–5.416; *P* = 0.004). Three-month mortality after discharged occurred more in frail patients than in non-frail patients, either defined by FRAIL or CFS (FRAIL: 31.9% vs. 14.9%, OR: 2.5; CI 95% 1.072–5.881; *P* = 0.034; CFS: 25.7% vs. 11.1%, OR: 3.7; 95% CI 1.255–10.812; *P* = 0.018). However, we did not find significant relation of the other negative outcomes, as shown in the Table 2.

**Table 2:** Predictive ability of the frailty instruments for adverse outcomes: multivariate analysis adjusted for sex, age, and principal diagnosis

	OR (CI 95%) associated with IF-VIG scale ≥ 0.2 (frail)	OR (CI 95%) associated with FRAIL scale ≥ 3 (frail)	OR (CI 95%) associated with Clinical Frailty Scale ≥ 4 (frail)	OR (CI 95%) associated with low^b^ hand grip strength (frail)
In-hospital mortality	1.142 (0.115–11.359)	1.620 (0.265–9.896)	3.029 (0.292–31.468)	2.028 (0.095–43.324)
Length of stay ≥ 6	1.518 (0.588–3.920)	2.739 (1.385–5.416)^•^	1.558 (0.748–3.246)	1.122 (0.468–2.687)
Functional decline	1.753 (0.398–7.731)	0.891 (0.347–2.289)	1.444 (0.484–4.307)	2.039 (0.380–10.950)
30-Days readmission	1.608 (0.562–4.601)	1.297 (0.641–2.626)	0.932 (0.431–2.017)	0.852 (0.345–2.100)
3-Month mortality	1.111 (0.362–3.408)	2.511 (1.072–5.881)^•^	3.684 (1.255–10.812)^•^	3.780 (0.973–14.692)
New institutionalization	0.366 (0.036–3.761)	0.619 (0.124–3.097)	0.217 (0.038–1.245)	3.737 (0.196–71.245)
Composite outcome^a^	1.645 (0.624–4.338)	1.590 (0.868–2.915)	1.626 (0.765–3.458)	1.391 (0.586–3.304)

^•^*P* value < .05

^a^Composite outcome: length of stay ≥ 6 or functional decline or new institutionalization

^b^Men with a hand grip strength < 23 kg and < 12 kg in women were considered frail

None of the frailty instruments resulted to be good predictors for any of the adverse outcomes (i.e. AUC-ROC above 0.7), as shown in Table 3 and Supplementary Fig. 2.

**Table 3:** The area under the receiver operating characteristic curves for VIG, FRAIL, clinical frailty scale and hand grip strength in predicting adverse outcomes

	IF-VIG scale	FRAIL scale	Clinical Frailty Scale	Hand grip strength^b^
	AUC (95% CI)	AUC (95% CI)	AUC (95% CI)	AUC (95% CI)
In-hospital mortality	0.659 (0.440–0.877)	0.619 (0.428–0.811)	0.568 (0.357–0.778)	0.643 (0.350–0.937)
Length of stay ≥ 6	0.553 (0.470–0.635)	0.591 (0.509–0.673)	0.562 (0.479–0.645)	0.520 (0.417–0.623)
Functional decline	0.548 (0.444–0.651)	0.550 (0.440–0.660)	0.617 (0.524–0.709)	0.570 (0.446–0.694)
30-Day readmission	0.508 (0.419–0.596)	0.507 (0.418–0.596)	0.508 (0.420–0.597)	0.517 (0.406–0.627)
3-Month mortality	0.627 (0.534–0.719)	0.644 (0.554–0.734)	0.666 (0.576–0.757)	0.563 (0.445–0.681)
New institutionalization	0.603 (0.421–0.784)	0.622 (0.471–0.773)	0.623 (0.410–0.836)	0.646 (0.437–0.856)
Composite outcome^a^	0.529 (0.445–0.614)	0.554 (0.469–0.638)	0.534 (0.450–0.618)	0.523 (0.420–0.625)

^a^Composite outcome: length of stay ≥ 6 or functional decline or new institutionalization

^b^Hand grip strength was distributed by sex

## Discussion

We present a prospective cohort study of patients admitted to an AGS with a median age of almost 90 years aiming to evaluate the accuracy, feasibility and predictive ability of four different frailty instruments. Prevalence of frailty according to the different instruments varied from 62.2 to 86.7%. Therefore, FRAIL, HGS and CFS showed a variable prevalence and an acceptable ability to detect frailty when referenced with the VIG instrument. In addition, the feasibility of the instruments ranged from 67 to 100%. Regarding the predictive ability of the instruments, a frail patient by FRAIL and CSF was 2.5 and 3.7 times more likely to die at 3 months, respectively, than a non-frail. Moreover, patients classified as frail by FRAIL were more likely to stay in the hospital for more than 6 days. However, being classified as frail by any of the frailty instruments was not associated with in-hospital mortality, institutionalization or readmission.

Frailty instruments must be selected according to the characteristics of the setting the setting where they are subministered, the ability to complete the test and the time required to perform it [25]. In this sense, we apply three brief instruments i.e. CFS, FRAIL and HGS (90, 24 and 90–120 s, respectively) and one longer i.e. VIG (10 min) in our study to measure frailty [6, 26]. Therefore, the first finding worth mentioning is the high feasibility of the self-reported instruments chosen, i.e., FRAIL, CFS or VIG in our AGS, in contrast to those containing objective measures such as the HGS. This different feasibility found between these two types of instruments is similar to the one found in the study by Oviedo-Briones et al. in patients admitted to geriatric wards [6].

On the other hand, the prevalence of frailty reported in our study is higher than the 20–50% reported in most of the studies [27–29], but it is nevertheless in line with that demonstrated by Chong et al. with a sample of patients very similar to ours, i.e. very old patients with comorbidity burden, low baseline functional status, high prevalence of cognitive impairment and a considerable proportion of patients coming from nursing homes [3, 30]. Regarding the ability of frailty instruments to predict mortality [14, 31, 32], we should emphasize that in patients admitted to AGS, decisions about diagnosis, treatment, and intervention are often made without a strong evidence base. Therefore, some patients may be subjected to overtreatment or adverse effects of interventions that cause distress at the end of their lives. In contrast, other patients who would potentially benefit from the intervention may not undergo these interventions only because of their advanced age. Hence, the ability to predict three-month mortality through FRAIL or CFS makes them useful tools for making tailored decisions in this setting. Moreover, the association between frailty according to the FRAIL and prolonged length of stay, in line with recent studies may also indicate that this scale adequately reflects the complexity of these patients in our sample [33]. With respect to HGS, it has been associated with functional status, mobility or mortality. However, our results do not support this correlation [7, 34]. A possible explanation for this lack of relation, could be that the population included in previous studies was younger and with better functional status than ours. On the other hand, the low feasibility of the test meant that we lost statistical power and excluded patients with a higher risk of mortality or functional impairment (i.e., those with more severe disease and worse functional status). With regards the capacity of detect frailty of the three instruments when referenced with the Spanish validated cumulative deficit approach instrument, we found that all of them were almost acceptable, but variable. One likely explanation is that this variability reflects that different scales are measuring different components of the construct of frailty.

Regarding the limitations of the study, we should highlight that we selected the VIG instrument as a reference, because it was validated to predict survival at 1 and 2 years in a sample similar to ours. However, as we have demonstrated in this study, the instrument is not adequate for predicting adverse outcomes among hospitalized older adults or three-month mortality [8, 19]. Another limitation of the present study is the small sample size, which means that our results must be interpreted with caution and need to be reproduced in other populations. In addition, the scales applied assess previous frailty and not that acquired during acute admission (except for the assessment of HGS on admission). This assessment at a different time and in different circumstances limits the comparison of their discriminative ability. Finally, we should highlight as a limitation of the study that we canceled the follow-up, due to the mortality impact that COVID-19 could cause in a sample in which 40% of the patients came from nursing homes. In addition, the low in-hospital mortality we found probably prevented us from finding a valuable instrument to predict it.

Regarding the strengths of the study, first we emphasize that it was conducted in an AGS through validated instruments, given that most of the studies which focused on frailty are conducted in non-geriatric disciplines and two thirds of them identify participants as frail even without measuring frailty [14]. Second, we have evaluated and compared within the same population three important characteristics of a frailty instrument should present i.e. accuracy, feasibility and predictive ability to be applied.

Therefore and according our results we can recommend to administer FRAIL and CFS in geriatric wards due to their high feasibility, accuracy to detect frail population and their ability predict short adverse outcomes as 3-month mortality. In contrast and according to our outcomes VIG and HGS are not highly recommendable in this setting. However, more researches are needed to be conducted in AGS to find better instruments to predict in-hospital mortality, institutionalization or readmission. In this sense finding frailty biomarkers as reference to measure frailty in the future, may allow to find an accurate clinical instrument of measurement [35].

## Conclusion

The prevalence of frailty in a sample of older patients admitted to an AGS according to different validated instruments is very high and varies from 63.2% to 74.6% when assessed using FRAIL and CFS, respectively. The CFS predicted mortality at three months and the FRAIL scale as well as three-month mortality was associated with prolonged length of stay (≥ 6 days). The full feasibility of both scales together with their ability to predict short-term adverse outcomes make them recommendable in this setting. VIG and HGS were not associated with any outcome. Finally, none of the instruments were related to in-hospital mortality, 30-day readmission and functional decline, warranting further studies with any other validated instrument in this setting to predict these outcomes.

## Supplementary Information

Below is the link to the electronic supplementary material.

Supplementary file1 (PDF 634 KB)

Supplementary file2 (PDF 672 KB)

Supplementary file3 (DOCX 20 KB)
