# Supplementary material for: Accuracy, feasibility and predictive ability of different frailty instruments in an acute geriatric setting
Source: Eur Geriatr Med. 2022 Apr 23;13(4):827–35. doi: 10.1007/s41999-022-00645-1 (PMC9034644; doi:10.1007/s41999-022-00645-1)
Supplement: Supplementary file 1 — Supplementary file1 (PDF 634 KB) [file 41999_2022_645_MOESM1_ESM.pdf]

SUPPLEMENTARY FIGURE 1: CLINICAL FRAILTY SCALE

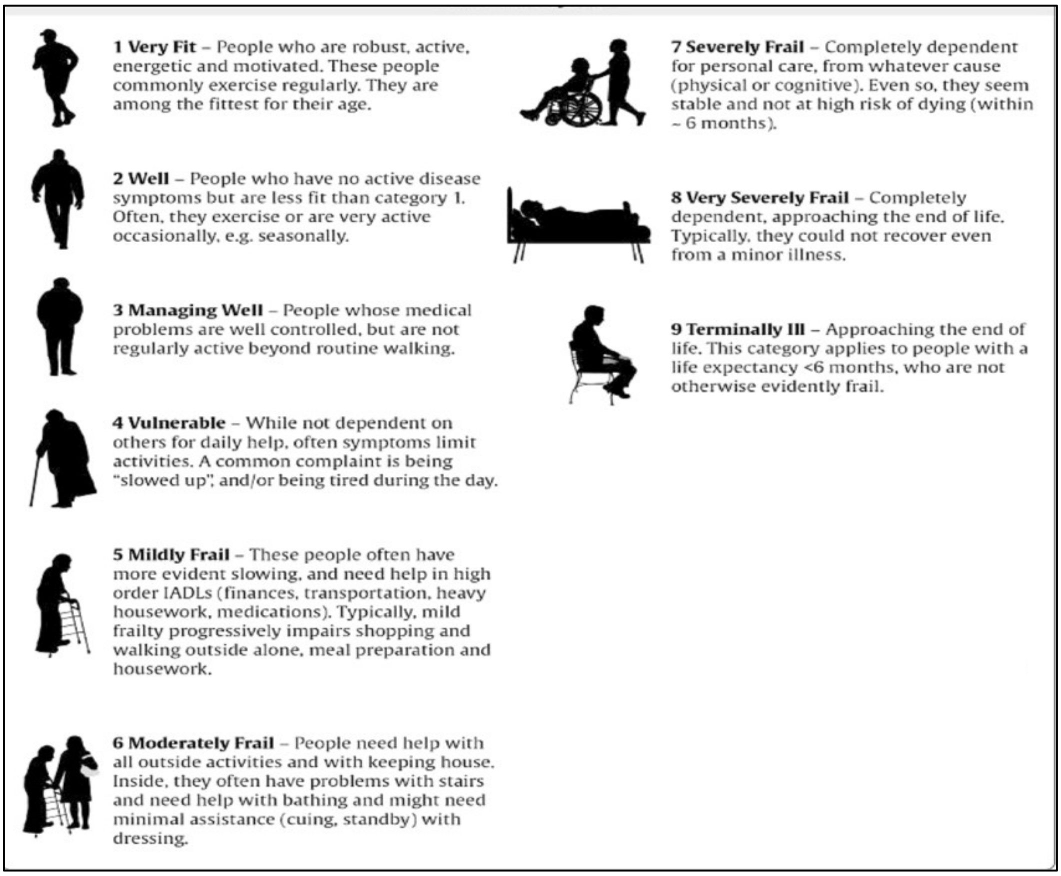

1 Very Fit – People who are robust, active, energetic and motivated. These people commonly exercise regularly. They are among the fittest for their age.

2 Well – People who have no active disease symptoms but are less fit than category 1. Often, they exercise or are very active occasionally, e.g. seasonally.

3 Managing Well – People whose medical problems are well controlled, but are not regularly active beyond routine walking.

4 Vulnerable – While not dependent on others for daily help, often symptoms limit activities. A common complaint is being "slowed up", and/or being tired during the day.

5 Mildly Frail – These people often have more evident slowing, and need help in high order IADLs (finances, transportation, heavy housework, medications). Typically, mild frailty progressively impairs shopping and walking outside alone, meal preparation and housework.

6 Moderately Frail – People need help with all outside activities and with keeping house. Inside, they often have problems with stairs and need help with bathing and might need minimal assistance (cuing, standby) with dressing.

7 Severely Frail – Completely dependent for personal care, from whatever cause (physical or cognitive). Even so, they seem stable and not at high risk of dying (within ~ 6 months).

8 Very Severely Frail – Completely dependent, approaching the end of life. Typically, they could not recover even from a minor illness.

9 Terminally Ill – Approaching the end of life. This category applies to people with a life expectancy <6 months, who are not otherwise evidently frail.

When the score is > 4, the patient is considered frail
