# Supplementary material for: Accuracy, feasibility and predictive ability of different frailty instruments in an acute geriatric setting
Source: Eur Geriatr Med. 2022 Apr 23;13(4):827–35. doi: 10.1007/s41999-022-00645-1 (PMC9034644; doi:10.1007/s41999-022-00645-1)
Supplement: Supplementary file 2 — Supplementary file2 (PDF 672 KB) [file 41999_2022_645_MOESM2_ESM.pdf]

SUPPLEMENTARY FIGURE 2

Panel 1. Functional decline

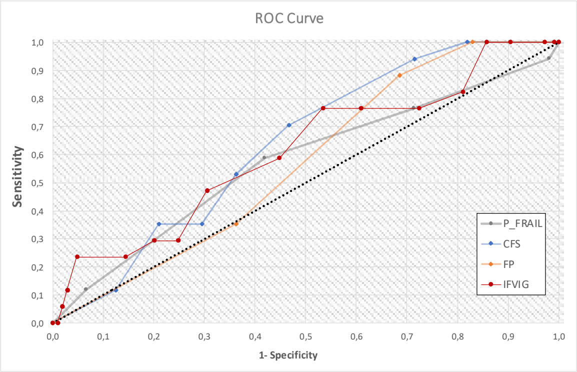

Frailty instrument	AUC (95% CI)	P-value
FRAIL	57.4 (41.8 - 72.9)	0.331
CFS	63.8 (51.9- 75.7)	0.068
HGS	53.4 (39.7 - 67.1)	0.655
VIG	61.1 (46.6 - 75.7)	0.141

Panel 2. Mortality

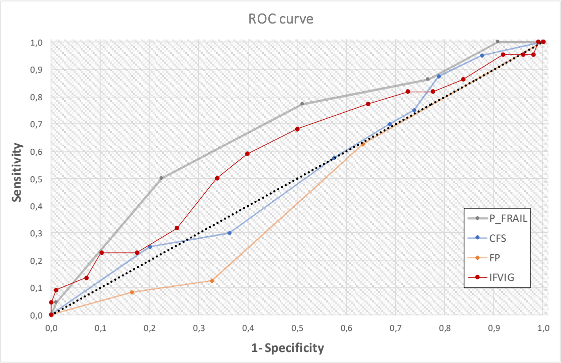

Frailty instrument	AUC (95% CI)	P-value
FRAIL	65.9 (53.9 - 78.0)	0.016
CFS	61.9 (49.8 - 74.0)	0.071
HGS	59.7 (46.6 - 72.7)	0.142
VIG	61.5 (48.7 - 74.3)	0.082

Panel 3. Readmission (30 days)

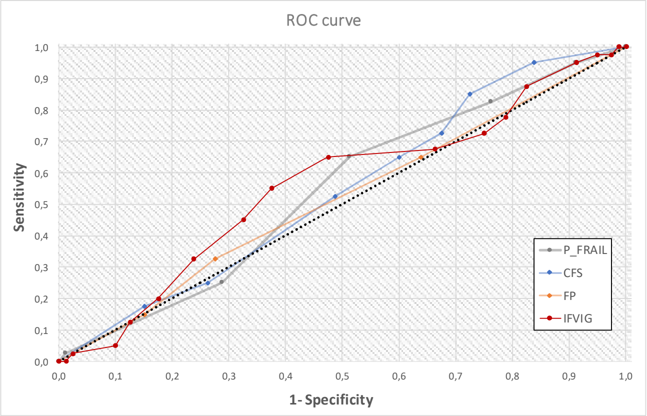

Frailty instrument	AUC (95% CI)	P-value
FRAIL	54.2 (43.5 - 64.9)	0.454
CFS	54.1 (43.5 - 64.7)	0.466
HGS	53.1 (42.2 - 64.0)	0.583
VIG	55.8 (44.8 - 66.9)	0.365

Supplementary figure 2. Area under the receiver operating characteristic curves for VIG, FRAIL, Hand Grip Strength (HGS) and Clinical Frailty Scale (CFS) in predicting adverse outcomes: Functional decline, Mortality and 30-days readmission
