# Supplementary material for: Accuracy, feasibility and predictive ability of different frailty instruments in an acute geriatric setting
Source: Eur Geriatr Med. 2022 Apr 23;13(4):827–35. doi: 10.1007/s41999-022-00645-1 (PMC9034644; doi:10.1007/s41999-022-00645-1)
Supplement: Supplementary file 3 — Supplementary file3 (DOCX 20 KB) [file 41999_2022_645_MOESM3_ESM.docx]

**SUPPLEMENTARY TABLE 1. FRAIL-VIG INSTRUMENT**

| Domain | Variable | Description |  | Points |
| --- | --- | --- | --- | --- |
| **Functional** | Money management | *Needs help managing financial matters (bank, shops, restaurants)* | Yes | 1 |
|  |  |  | No | 0 |
|  | Telephone use | *Needs help using the telephone* | Yes | 1 |
|  |  |  | No | 0 |
|  | Medication management | *Needs assistance in preparing or administering medications* | Yes | 1 |
|  |  |  | No | 0 |
|  | Barthel index (BI) | *Barthel index (BI) No dependency (BI ≥ 95)* |  | 0 |
|  |  | *Mild-moderate dependency (BI 90–65)* |  | 1 |
|  |  | *Moderate-severe dependency (BI 60–25)* |  | 2 |
|  |  | *Absolute dependency (BI ≤ 20) 3* |  | 3 |
| **Nutritional** | Malnutrition | *Weight loss ≥ 5% in the last 6 months* | Yes | 1 |
|  |  |  | No | 0 |
|  |  | *No cognitive impairment* |  | 0 |
| **Cognitive** | Degree of cognitive impairment | *Mild-moderate cognitive impairment (equivalent to GDS ≤ 5)* |  | 1 |
|  |  | *Severe-very severe cognitive impairment (equivalent to GDS ≥ 6)* |  | 2 |
| **Emotional** | Depressive syndrome | *Need for antidepressant medication* | Yes | 1 |
|  |  |  | No | 0 |
|  | Insomnia/anxiety | *Frequent need for benzodiazepines or other psychiatric drugs with a sedative effect for* | Yes | 1 |
|  |  | *insomnia/anxiety* | No | 0 |
|  |  |  |  |  |
| **Social** | Social vulnerability | *Do health care professionals perceive the presence of social vulnerability?* | Yes | 1 |
|  |  |  | No | 0 |
|  | Delirium. | *Presence of delirium and/or behaviour disorder requiring antipsychotic drugs in the last 6 months.* | Yes | 1 |
| **Geriatric syndromes** |  |  | No | 0 |
|  | Falls | *In the last 6 months, ≥2 falls or hospitalization due to a fall.* | Yes | 1 |
|  |  |  | No | 0 |
|  | Ulcers | *Presence of ulcer (pressure or vascular, any grade)* | Yes | 1 |
|  |  |  | No | 0 |
|  | Polypharmacy | *Taking ≥ 5 drugs* | Yes | 1 |
|  |  |  | No | 0 |
|  | Dysphagia | *Difficulty swallowing when eating or drinking? Presence of aspiration respiratory infections during the last 6 months?* | Yes | 1 |
|  |  |  | No | 0 |
| **Severe symptoms** | Pain | *Need for ≥ 2 conventional analgesics and/or strong opioids for pain control* | Yes | 1 |
|  |  |  | No | 0 |
|  | Dyspnea | *Basal dyspnea impeding the ability to leave the house and/or opioids are frequently needed* | Yes | 1 |
|  |  |  | No | 0 |
| **Diseases (+)** | Cancer | *Active cancer* | Yes | 1 |
|  |  |  | No | 0 |
|  | Respiratory | *Presence of any type of chronic respiratory disease (COPD, restrictive lung disease...)* | Yes | 1 |
|  |  |  | No | 0 |
|  | Cardiac | *Presence of any type of chronic heart disease (heart failure, ischemic cardiomyopathy, arrhythmia)* | Yes | 1 |
|  |  |  | No | 0 |
|  | Neurological | *Presence of any type of neurodegenerative disease (Parkinson, ALS,...) or a history of stroke (ischemic or hemorrhagic).* | Yes | 1 |
|  |  |  | No | 0 |
|  | Digestive | *Presence of any type of chronic digestive disease (chronic liver disease, cirrhosis, chronic, pancreatitis, inflammatory bowel disease,…)* | Yes | 1 |
|  |  |  | No | 0 |
|  | Renal | *Presence of chronic renal failure (GFR < 60)* | Yes | 1 |
|  |  |  | No | 0 |
|  |  | **FRAIL VIG INDEX** | | **X/25** |

*(+) two point are scored if the patient presents criteria for advanced chronic illness on the NECPAL*

*Available at: http://ico.gencat.cat/web/.content/minisite/ico/professionals/documents/qualy/arxius/NECPAL-3.0-ENGLISH_full-version.pdf)*

*When the VIG scores is ≥ 0.2 the patient is considered frail*

**SUPPLEMENTARY TABLE 2. FRAIL INSTRUMENT**

| **1) Fatigue** |
| --- |
| How much of the time during the past 4 weeks did you feel tired? |
| 1 = All of the time, 2 = Most of the time, 3 = Some of the time, 4 = A little of the time, 5 = None of the time. |
|  |
| Responses of “1” or “2” are scored as 1 and all others as 0. |
|  |
| **2) Resistance** |
| By yourself and not using aids, do you have any difficulty walking up 10 steps without resting? |
|  |
| 1 = Yes, 0 = No. |
|  |
| **3) Ambulation** |
| By yourself and not using aids, do you have any difficulty walking a couple of blocks (e.g. several hundred yards)? |
|  |
| 1 = Yes, 0 = No. |
|  |
| 4) **Illnesses** |
| Did a doctor ever tell you that you have [illness]? How many (see list below): |
| The total illnesses (0–11) are recoded as 0–4 = 0 and 5–11 = 1. The illnesses include hypertension, diabetes, cancer (other than a minor skin cancer), chronic lung disease, heart attack, congestive heart failure, angina, asthma, arthritis, stroke, and kidney disease. |
|  |
| 5) **Loss of weight** |
| Have you lost at least 5% of your usual weight in the last year? |
| 1 = Yes, 0 = No. |
| *A score of 0 represents robust health status; 1-2: Pre-frail; 3-5: Frail* |
